# Prevalence of Carpal Tunnel Syndrome Symptoms Among Young Dentists

**DOI:** 10.7759/cureus.43358

**Published:** 2023-08-12

**Authors:** Zeliha Matur, Tunahan Zengin, Naci Emre Bolu, Ali Emre Oge

**Affiliations:** 1 Department of Neurology, Bezmialem Vakif University, Faculty of Medicine, Istanbul, TUR; 2 Department of Neurology, Istanbul University, Istanbul Faculty of Medicine, Istanbul, TUR; 3 Department of Internal Medicine, Trakya University, Faculty of Medicine, Edirne, TUR; 4 Department of Neurology, Maltepe University, Faculty of Medicine, Istanbul, TUR; 5 Departments of Neurology and Clinical Neurophysiolgy, Istanbul University, Istanbul Faculty of Medicine, Istanbul, TUR

**Keywords:** frequent object dropping, dentists, entrapment neuropathy, boston carpal tunnel syndrome questionnaire, carpal tunnel syndrome

## Abstract

Objectives

Although age is a risk factor, carpal tunnel syndrome (CTS) can also affect younger individuals, particularly those involved in activities or occupations that require repetitive hand movements, forceful gripping, or prolonged wrist flexion/extension. This case-control study aimed to examine the prevalence of CTS symptoms and frequent object dropping among a group of young dentists who are exposed to CTS risk factors. Additionally, other reported risk factors for CTS, such as sex, obesity, and square wrist sign, were also investigated.

Methods

A total of 74 dentists (48 women, mean age 28.5 years), who are working at Istanbul Faculty of Dentistry, the largest dental school in Istanbul, which is the biggest city in Turkey, were included in the study. Additionally, 61 age- and sex-matched controls (38 women, mean age 27.9 years) were also recruited. The Edinburgh Hand Preference Questionnaire, Boston Carpal Tunnel Syndrome Questionnaire (BCTQ), a questionnaire for object dropping and occupational hand usage, anthropometric measurements of the hands, clinical neurologic examination, and electromyography intended for the detection of CTS were performed.

Results

The dentists had a higher total weekly hand usage duration compared to the controls (66.3 vs 44.8 hours, p<0.001). BCTQ scores and the frequency of object dropping were also significantly higher in dentists compared to controls (respective p values: 0.011, 0.003). Positive correlations were found between BCTQ scores, hand usage durations, and object dropping (respective p values: 0.001, <0.001). BCTQ scores were higher in women than in men (p=0.027). Electrophysiologic evidence of CTS was found in one dentist.

Conclusions

Symptoms of CTS may manifest in individuals at a younger age than predicted, primarily influenced by their occupation and the duration of hand usage. Dentists, in particular, report a higher incidence of complaints related to object dropping, which can be attributed to their frequent use of specialized tools and engagement in delicate tasks, resulting in heightened awareness. However, it can also potentially serve as an indicator of CTS.

## Introduction

Carpal tunnel syndrome (CTS) is the most common entrapment neuropathy, caused by an increase in tissue pressure and compression in the carpal tunnel. Its overall prevalence in the general population is around 5% [1,2]. CTS is more common in women, older adults, and white individuals. The likelihood of developing CTS increases with age due to various factors such as age-related degenerative changes in the wrist, decreased tissue elasticity, and cumulative wear and tear on the median nerve [1-3]. Additionally, conditions such as arthritis or diabetes, which are more common in older adults, can further increase the risk of developing CTS [2,3]. CTS can still occur in younger individuals, especially if they engage in activities or occupations that involve repetitive hand movements, forceful gripping, or prolonged wrist flexion/extension [3-5].

Many occupations and work activities carry a risk of developing CTS due to their demanding repetitive hand movements, forceful gripping, or prolonged wrist flexion and extension. For instance, workers in manufacturing execute repetitive tasks that necessitate constant hand and wrist motions. Construction workers frequently engage in forceful gripping or prolonged wrist movements. Musicians, especially those playing string or percussion instruments, also experience these repetitive movements. Data entry operators repetitively use keyboards, which involve wrist flexion and extension. Hairstylists and barbers regularly employ scissors and clippers that require repetitive hand movements. Additionally, chefs and cooks often perform repetitive actions such as chopping or stirring [4,5]. Dentists are also significantly involved in precision work with their hands and they are at risk for developing CTS due to the repetitive wrist movements and increased pressure in the palm during dental procedure [6,7].

Numbness, tingling, and prickling in the digits are the most common symptoms of CTS. An increase in pain and numbness at night, which can interrupt sleep and can be relieved by shaking hands, is typical. Weakness in the hands and eventual atrophy in the thenar eminence are more commonly seen in advanced cases [2,8]. Patients may experience difficulty holding and gripping objects, making a fist, and using smaller objects. Some individuals may also report a feeling of swelling in their fingers [8]. In some patients with CTS, there is a complaint of frequent object dropping. This symptom has been reported to be more common in women and the elderly and is related to the severity of CTS [9].

The complaint of frequent object dropping may be primarily associated with diseases that cause tremors, muscle weakness, sensory deficits, and motor coordination impairment. However, it is important to note that dropping objects can also occur for other reasons, such as clumsiness or distraction, and may not necessarily be a symptom of a medical condition. This distressing symptom can also be seen in some patients with CTS. In patients with CTS, the increased frequency of paraesthesia, numbness, and weakness is found to be related to frequent object dropping, but it is unclear whether motor or sensory nerve fiber involvement has a greater impact on this symptom [10].

It is known that obesity is an important risk factor for CTS. Studies have reported that obese individuals have a 1.5 times higher risk of developing CTS, and each 1-point increase in body mass index (BMI) can lead to an 8% increase in CTS risk [2,4]. The size and shape of the carpal tunnel can play a role in the development of CTS. The wrist ratio is calculated by dividing the wrist depth (anterior-to-posterior dimension) by the wrist width (medial-to-lateral dimension) and is measured in millimeters. When the ratio is close to 1 (1:1), meaning the depth and width are approximately equal, it is considered a square wrist sign. A square wrist sign may be indicative of a narrower carpal tunnel, and in some cases, it has been associated with an increased risk of developing CTS [11]. However, it is important to remember that the square wrist sign alone is not a definitive diagnostic method for CTS, and it is typically used in conjunction with other clinical evaluations and tests. In conclusion, the wrist ratio or square wrist sign provides valuable information about wrist dimensions and can be considered as one of the factors when assessing the risk of developing CTS [11].

As mentioned before, certain occupational groups pose risk factors for CTS, and symptoms of CTS may start earlier than expected within these professional groups. Building upon this hypothesis, this study aims to investigate the presence of CTS symptoms in a professional group at an age range earlier than the typical onset age of this syndrome. Dentistry was chosen as the focus due to its known association with increased susceptibility to CTS [5,6]. The use of dental instruments requires precise and repetitive movements of the wrist and fingers, which can lead to irritation and inflammation of the tissues in and around the wrist, ultimately resulting in CTS. Additionally, the pressure applied to the palm during dental procedures can also contribute to the development of CTS [3,5,6]. A control group with a similar age and sex distribution, but not requiring occupational tool use, was compared to dentists. According to our information, the presence of frequent object-dropping complaints has not been investigated before in a group of individuals who use their hands professionally for long periods of time and who did not have clinically or electrophysiologically evident CTS. Therefore, this study also investigated the relationship between frequent object dropping and CTS symptoms. Furthermore, other reported risk factors for CTS, such as sex, obesity, and the square wrist sign, were also examined.

This article was previously presented as a meeting abstract at the 16th European Congress of Clinical Neurophysiology (ECCN), on August 30 to September 2, 2017, in Budapest, Hungary.

## Materials and methods

This study was designed as a case-control study and approved by the Istanbul University Faculty of Medicine Ethical Committee (number: 2015/234). Subjects were included in the study after they gave their informed consent.

Recruitment of the subjects

The dentists who participated in this study were selected among those working at Istanbul University Faculty of Dentistry, which is situated on the same campus as Istanbul University Istanbul Faculty of Medicine, located in Istanbul, the largest city in Turkey with its dense population. The interviews were conducted with dentists, under the age of 40, working in three specific departments (Mouth, Tooth, and Jaw Surgery; Orthodontics; and Periodontology) out of a total of eight departments in the Faculty of Dentistry. The dentists were serving as assistants, specialists, or associate professors. All of them agreed to participate. Seventy-nine volunteers in the age range of 23-40 were interviewed and individuals with any disease that may affect the central and /or peripheral nervous system were excluded. Finally, 74 healthy dentists were included. The control group consisted of administrative personnel working at the hospital whose job roles did not involve extensive hand usage and their age and sex distributions were matched to those of the dentists. The subjects included in the control group had no known diseases or history of medication use, and they did not have any diagnosis of entrapment neuropathy, including CTS.

Application of the survey

Each subject filled out the Edinburgh Handedness Inventory questionnaire [12]. The questionnaire consisted of 10 activities (ranging from “writing with a pencil” to “opening a jar”) to score, and five choices for each of these activities (always left=-10, usually left=-5, no preference=0, usually right=+5, always right=+10). We calculated the total of the scores given and concluded that they were left-handed if the score was below -25 and right-handed if the score was higher than +25. If the total was between -25 and +25, we concluded the participant was ambidextrous.

Then they filled out the Boston Carpal Tunnel Syndrome Questionnaire (BCTQ) [13]. The Turkish version's functionality and reliability were assessed by Sezgin et al. BCTQ, a validated questionnaire (see Appendix), was used to evaluate the severity and functional impact of symptoms related to CTS [14]. It consists of two parts: Symptom Severity Scale (SSS) and the Functional Status Scale (FSS). SSS assesses the severity of CTS symptoms, including pain, numbness, tingling, and weakness in the hand and fingers. It consists of 11 questions asking the respondent to rate the severity of their symptoms on a scale from 1 (mild) to 5 (severe). FSS measures the functional limitations caused by CTS. It consists of eight questions asking the respondent to rate their ability to perform various daily activities, such as writing, gripping objects, and opening jars, on a scale from 1 (no difficulty) to 5 (unable to do). Higher scores are considered to imply a more severe CTS.

**Figure 1:**
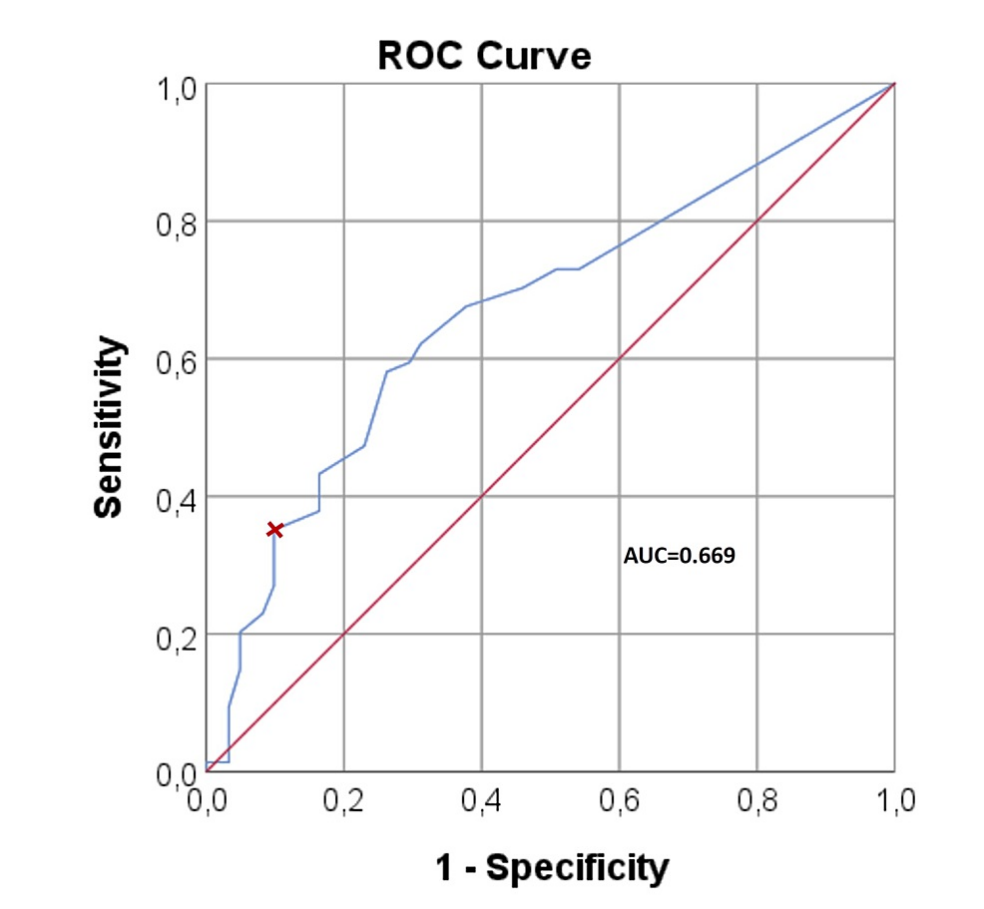
The receiver operating characteristic (ROC) analysis plot. The area under the ROC curve (AUC) is close to 0.5, indicating that it doesn't discriminate dentists from controls. BCTQ-total >30 identifies the dentists with 90% specificity and 35% sensitivity. BCTQ: Boston Carpal Tunnel Syndrome Questionnaire

Since there were individuals in both groups with BCTQ scores higher than the minimum possible values they could obtain (specifically, 11 for BCTQ-SSS, eight for BCTQ-FSS, and 19 for the BCTQ-total), a receiver operating characteristic (ROC) analysis was conducted in an attempt to identify a threshold score that could differentiate dentists from the control group. On the plotted ROC curve (Figure 1) dentists and controls could not be effectively distinguished based on the BCTQ scores. When the BCTQ-total score exceeded 30, subjects were classified into the dentist group with 90% specificity and 35% sensitivity (the area under the ROC curve was 0.669).

Clinical examination and anthropometric measurements of hands

All subjects underwent a brief neurological examination (by Z.M. and A.E.O.) dedicated to CTS. Both hands were investigated for sensory deficits, muscle weakness, presence of atrophy, and positivity of Phalen’s test. For paraesthesia, the subjects were questioned about feelings of tingling, numbness, burning, and pricking in their hands; their frequency and the time of the day they occur, their duration, and whether or not they were relieved by shaking the hands. Hypoesthesia was determined by rubbing a single cotton to the medial and lateral borders of the fifth and secondfingers, respectively, while the subject’s eyes were closed, and asking if they felt a difference. For Phalen’s test, the subjects were asked to hold their wrists in complete and forced flexion (pushing the dorsal surfaces of both hands together) for 60 seconds and then questioned if they had felt tingling, numbness, or burning in their hands during or afterward.

Body weights and heights were recorded, and BMI values were calculated as weight in kilograms divided by height in meters squared. The anthropometric measurements of the hand were conducted only in the dentist group (by T.Z., N.E.B., and A.E.O.). For these measurements, wrist thickness (dorsal-palmar diameter), wrist width (lateral-medial diameter), and hand length were measured. The wrist thickness and wrist width were measured in millimeters with a caliper. These measurements were taken across the middle of the proximal and distal creases of the wrist, with the caliper being applied with mild pressure until there were slight skin indentations on both sides of the blades. The wrist ratio was calculated by dividing the wrist thickness by the wrist width [11,15]. Hand length was measured from the tip of the third digit to the distal crease of the wrist, using a fabric tape measure

Hand usage and dropping of object

The subjects were asked about the amount of time for their occupational hand usage, keyboard and smartphone use, hand-related hobbies, housework, and child care, as well as the frequency of pen/pencil use during a typical week. They were also asked if they had experienced any incidents of dropping objects (without external conditions that could cause the dropping). However, initial trial runs, which were not included in the study, revealed that people often have difficulty recalling how many times they dropped objects in the past three months when they were questioned retrospectively. Therefore, the subjects were asked to keep a diary to note as soon as possible after each incident of object dropping and they were contacted after three months to receive their reports about the total number of incidents during that period. Object dropping frequency was classified as rare (less than once a month), occasional (more than once a month but less than once a week), frequent (more than once a week but less than once a day), and very frequent (equal to or more than once a day).

Nerve conduction studies

Nerve conduction studies (NCSs) were performed using an electromyography device (Viking® on Nicolet® EDX; Natus Neurology Inc., Middleton, WI, USA) by one clinical neurophysiologist with 12 years of experience (Z.M.). Due to the demanding nature of their work schedules, most dentists were reluctant to undergo electromyography (EMG) examination. Therefore, individuals with BCTQ-total scores greater than the determined threshold value of 30 through ROC analysis were invited for further evaluation. Among the dentists, 26 individuals had BCTQ-total scores greater than 30, while in the control group, six individuals had scores exceeding 30.

Bilateral orthodromic median secondfinger and ulnar fifth finger sensory NCSs were conducted, along with median-ulnar ring finger sensory latency comparison. For the median sensory NCS, saddle-shaped recording electrodes were placed on the ventral aspect of the wrist, over the median nerve, typically 1.5-2 cm proximal to the proximal wrist crease, and stimulating ring electrodes were placed around the proximal (active) and middle (reference) phalanges on the second digit. For the ulnar sensory NCS, recording electrodes were placed on the ventral aspect of the wrist, over the ulnar nerve, typically about 1 cm proximal to the proximal wrist crease, and stimulating ring electrodes were placed around the proximal (active) and middle (reference) phalanges on the fifth digit. Onset latency, peak latency, peak-to-peak amplitude, and conduction velocity were measured for every sensory nerve action potential (SNAP). If the median nerve peak latency of over 3.2 ms was considered to be prolonged and the sensory nerve conduction velocity is below 50 m/s, it is considered slow. For median-ulnar sensory latency comparison, ring electrodes were used to stimulate the fourth finger, and recordings were made at the wrist on the median and ulnar nerves, keeping equal conduction distances. The difference between median and ulnar sensory peak latencies was measured, and if it was over 0.4 ms, it was considered abnormal [16]. During the examination, the temperature of the upper extremity was maintained at 32-34 °C.

The median and ulnar motor NCSs were performed bilaterally. For the median motor NCS, an active electrode was placed on the middle portion of the abductor pollicis brevis muscle, and stimulation was applied at the wrist and elbow. For the ulnar motor NCS, the active electrode was placed on the middle of the abductor digiti minimi muscle, and stimulation was applied at the wrist, below the elbow, and above the elbow. Onset latency, amplitude, and conduction velocities were measured. Median nerve onset latencies over 4.2 ms with wrist stimulation were considered as prolonged [16].

Statistical analysis

All analyses were performed in SPSS (version 25.0; IBM, Armonk, NY, USA). Descriptive statistics for continuous variables were presented as mean ± standard deviation (sd) and as median (interquartile range), categorical variables were given as count (%). The Kolmogorov-Smirnov test was used to check if a variable was normally distributed. Student’s T-test was used for comparing normally distributed continuous variables and the Mann-Whitney U test was used if the data were not normally distributed. Chi-Square Test was used for comparing categorical variables. Correlation analysis was performed by using Spearman's rank-order correlation test for non-parametric variables and by using Pearson correlation for parametric variables. To determine which variable(s) among those found to be correlated with BCTQ scores were effective on BCTQ scores, a multivariate analysis of variance (MANOVA) test was used.

Except for the Kolmogorov-Smirnov test used to test for normal distribution (one-tailed), other statistical analyses employed two-tailed tests. The significance level was set as <0.05. The ROC analysis was conducted to determine a threshold value that could distinguish between the two groups based on BCTQ scores.

## Results

The dentist group was comprised of 74 individuals with a median age of 28 years, of whom 48 (56%) were women. The control group consisted of 61 subjects with a median age of 26 years, including 38 (44%) women. Among the dentists, 64 individuals were right-handed (including three left-handed and seven ambidextrous), while in the control group, 55 individuals were right-handed (including two left-handed and four ambidextrous). In the dentist group, 8.1% (six dentists) had a family history of CTS, while in the control group, it was observed in 14.8% (nine subjects). There were no significant differences between the groups regarding age and sex distribution, hand preference, and family history of CTS. There were no significant differences between the groups in terms of height, weight, and BMI. The number of individuals with a BMI of 25-29.9 (overweight) was 18 in the dentist group and 10 in the control group. The number of individuals with a BMI of 30 and above (obese) was two in the dentist group and three in the controls.

Weekly occupational and total hand usage was significantly higher in the dentists than in the controls (Table 1). In the dentist group, 41 individuals (55.4%) reported dropping objects from their hands, while in the control group, 12 individuals (19.7%) reported similar complaints (p <0.001). The frequencies of object dropping in the dentist and control groups were as follows; rare: 11 to 6, occasional: 13 to 4, frequent: 13 to 2, very frequent: 4 to 0. Neurological examination did not reveal any muscle weakness or atrophy of the median nerve innervated thenar muscles. Dentists experienced paraesthesia in their hands, with 18 on the dominant side and 15 on the non-dominant side. Positive Phalen's sign was detected in both groups, with a higher prevalence observed among dentists (Table 2).

**Table 1:** Demographic data, duration of hand usage, and BCTQ scores in the groups. N: number of cases, sd: standard deviation, range: interquartile range, Test Stat: test statistics (*BMI showed a normal distribution, and T-Test was used for comparison, while others were compared using the Mann-Whitney U test), BMI: body mass index, BCTQ: Boston Carpal Tunnel Syndrome Questionnaire, SSS: symptom severity scale, FSS: functionality status scale.

	Dentists (N=74, 48 women)		Controls (N=61, 38 women)		Test Stat.	p
	Mean (sd)	Median (range)	Mean (sd)	Median (range)		
Age (years)	28.5 ± 3.8	28 (23 - 40)	27.9 ± 6.06	26 (20 - 44)	1860	0.078
Weight (kg)	66.7 ± 15	62 (45 - 118)	66.91 ± 14.22	65 (42 - 102)	1689	0.734
Height (m)	1.7 ± 0.1	1.7 (1.6 - 2)	1.68 ± 0.08	1.67 (1.55 - 1.88)	1302	0.069
BMI (kg/m^2^)	22.6 ± 3.3	21.8 (18 - 31)	23.59 ± 3.95	23.34 (17 - 33)	1905*	0.123
Occupational Hand Usage (hours/week)	34 ± 13.7	33.5 (3 - 60)	20.43 ± 15.77	15 (0 - 65)	1178	<0.001
Total Hand Usage (hours/week)	66.3 ± 22.5	67 (22 - 100)	44.8 ± 23.76	42 (6 - 110)	1153	<0.001
BCTQ-SSS	15.1 ± 4.3	15 (11 - 33)	13.41 ± 3.14	12 (11 - 22)	1716	0.014
BCTQ-FSS	12.1 ± 3.7	11.5 (8 - 25)	9.74 ± 2.98	8 (8 - 22)	1307	<0.001
BCTQ-Total	27.2 ± 6.9	27 (19 - 45)	23.15 ± 5.64	21 (19 - 41)	1494	0.001

**Table 2:** Neurological examination findings obtained in the groups. N: number of cases, Test Stat: test statistic (Pearson chi-square test), D: dominant side, ND: Non-dominant side.

Symptom or sign	Groups		Test Stat.	p
	Dentists (N=74)	Controls (N=61)		
Paraesthesia in the hands				
D	18 (24.7%)	0	17.4	<0.001
ND	15 (20.5%)	0	14.1	<0.001
Hypoesthesia in the hands and fingers				
D	0	0		
ND	0	0		
Weakness of median innervated hand muscle				
D	0	0		
ND	0	0		
Phalen’s sign				
D	11 (15.1%)	3(4.9%)	3.6	0.059
ND	7 (9.6%)	3(4.9%)	1.0	0.316

In the dentist group, 54 individuals reported symptoms and functional impairment related to CTS according to the BCTQ, while in the control group, this was observed in 33 individuals. In the dentists, BCTQ scores were also significantly higher compared to the control group (Table 1). When evaluated in terms of the presence of Phalen's sign, which could be regarded as an objective examination finding for CTS, it was observed that subjects with a positive Phalen's sign had longer occupational hand use durations (p=0.046). BCTQ scores were higher in individuals with a positive Phalen's sign compared to those without: median values were 19 to 12 for BCTQ-SSS (p=0.001), 13 to 9 for BCTQ-FSS (p=0.004), and 33 to 23 for total BCTQ (p=0.001).

The results of anthropometric measurements applied to the hands of dentists are presented in Table 3. Except for wrist ratios, men’s measurements were higher than women’s in every category. On the dominant side, the average hand length was 17.5 cm for women and 19.5 cm for men. The mean wrist thickness and wrist width on the dominant side were 35.9 mm and 50.3 mm for women, respectively, while for the males, they were measured as 41.5 mm and 57.5 mm. The wrist ratios were similar in women (dominant hand: 0.71±0.06), and men (dominant hand: 0.72±0.04). There was no significant difference between the dominant and non-dominant hands in terms of anthropometric hand measurements.

**Table 3:** Anthropometric measurements of hands in dentists. N: number of cases, sd: standard deviation, range: interquartile range, D: dominant side, ND: non-dominant side. * Wrist thickness/wrist width

	Men (N=26)		Women (N=48)			Total (N=74)	
	Mean (sd)	Median (range)	Mean (sd)	Median (range)	P value*	Mean (sd)	Median (range)
Wrist diameters (mm)							
Wrist thickness D	41.5 (3)	41.6 (10.5)	35.9 (2.8)	35.3 (15.6)	<0.001	37.9 (4)	37.1 (15.6)
ND	41.3 (3.2)	41.4 (13.3)	35.6 (2.8)	35 (16.7)	<0.001	37.6 (4)	36.6 (16.7)
Wrist width D	57.5 (3.6)	57.9 (15)	50.3 (2.9)	50 (10.8)	<0.001	52.8 (4.7)	51.8 (20.7)
ND	57.2 (3.4)	57.3 (12.1)	50.4 (3)	49.8 (11.6)	<0.001	52.8 (4.5)	51.8 (17.2)
Hand length (cm)							
D	19.5 (1.3)	19.5 (5)	17.5 (0.8)	17.5 (4)	<0.001	18.2 (1.4)	18 (6.5)
ND	19.6 (1.3)	19.5 (5)	17.5 (0.8)	17.5 (3.5)	<0.001	18.2 (1.4)	18 (6.5)
Wrist Ratio*							
D	0.7 (0)	0.7 (0.2)	0.7 (0.1)	0.7 (0.4)	0.308	0.7 (0.1)	0.7 (0.4)
ND	0.7 (0)	0.7 (0.2)	0.7 (0.1)	0.7 (0.4)	0.097	0.7 (0.1)	0.7 (0.4)

The subjects with BCTQ-total scores above 30 who accepted the examination underwent EMG testing (20 dentists and four controls). Electrophysiological examinations directed to the diagnosis of CTS were found to be normal in all dentists and controls except one women dentist who was diagnosed with minimal CTS according to Padua classification [17]. She was 27 years old and her BMI was 25.4. There was a positive Phalen’s sign on her dominant hand, and her BCTQ-total score was 36. She reported the frequency of dropping objects as more than once a week. She was followed up with splint treatment for one year. Her symptoms and EMG findings improved. No significant differences were found between the two groups in terms of the mean median and ulnar sensory and motor nerve conduction values (Table 4).

**Table 4:** Nerve conduction studies. N: number of cases, sd: standard deviation, range: interquartile range, Test Stat: test statistic (Mann-Whitney U), D: dominant side, ND: non-dominant side, PL: peak latency, Diff. median-ulnar 4th PL: difference between the median and ulnar fourth digit peak latency.

	Latency (ms)	Dentists (N=20, 14 women)		Controls (N=4, 3 women)		Test Stat.	p
Median Nerve		Mean (sd)	Median (range)	Mean (sd)	Median (range)		
Sensorial	2^nd ^digit PL						
	D	2.8 (0.2)	2.7 (0.8)	2.8 (0.1)	2.8 (0.3)	34	0.640
	ND	2.7 (0.2)	2.7 (0.8)	2.7 (0)	2.7 (0.1)	37	0.816
	Diff. median-ulnar 4^th^ digit PL						
	D	0.1 (0.1)	0.1 (0.5)	0.1 (0.1)	0.1 (0.2)	33.5	0.613
	ND	0.1 (0.1)	0.1 (0.5)	0.1 (0.1)	0.1 (0.2)	27	0.310
Motor	Distal latency						
	D	2.9 (0.3)	2.9 (1.2)	2.9 (0.3)	2.9 (0.5)	37.5	0.968
	ND	2.8 (0.3)	2.9 (1)	2.8 (0.3)	2.7 (0.7)	27	0.530

Correlation analyses were performed between BCTQ scores and some clinical parameters (Table 5). There were positive correlations between BCTQ scores and the frequency of object dropping and hand usage duration. However, interestingly, a negative correlation was found between BCTQ scores and body weight and BMI. The BCTQ-SSS and BCTQ-total scores were significantly higher in women compared to men (p=0.22, p=0.027, respectively). There was no correlation between the BCTQ scores and anthropometric measurements of the hand.

**Table 5:** The factors associated with BCTQ scores. BCTQ: Boston Carpal Tunnel Syndrome Questionnaire, SSS: symptom severity scale, FSS: functional status scale, BMI: body mass index, D: dominant, ND: non-dominant.

	BCTQ-SSS		BCTQ-FSS		BCTSQ- Total	
	Correlation Coefficient	p	Correlation Coefficient	p	Correlation Coefficient	p
Frequency of object dropping	.206	0.017	.276**	0.001	.250**	0.003
Occupational hand usage (hours/week)	.239	0.005	.227**	0.008	.261**	0.002
Total activity (hours/week)	.142	0.001	.252**	0.003	.218*	0.011
Height (m)	-0.092	0.321	-0.044	0.633	-0.064	0.494
Weight (kg)	-.189	0.041	-0.174	0.059	-.187*	0.043
BMI (kg/m^2^)	-.209	0.023	-.213*	0.021	-.224*	0.015
Wrist thickness D	-0.109	0.355	-0.137	0.243	-0.122	0.299
Wrist thickness ND	-0.127	0.283	-0.174	0.14	-0.144	0.225
Wrist width D	-0.206	0.078	-0.209	0.073	-0.206	0.078
Wrist width ND	-0.18	0.126	-0.17	0.149	-0.166	0.157
Hand length D	-0.039	0.747	-0.105	0.381	-0.065	0.59
Hand length ND	-0.034	0.776	-0.082	0.491	-0.047	0.69

A MANOVA test was conducted with the dependent variable being the BCTQ scores and the independent variables being group, gender, BMI, frequent object dropping, and total hand usage time (total activity) (Table 6). Being a dentist showed a significant effect on BCTQ scores, with statistically significant p-values observed for BCTQ-SSS, BCTQ-FSS, and BCTQ-total (p=0.021, p=0.008, and p=0.004, respectively). Moreover, the combination of being a dentist and having frequent object-dropping complaints had a noteworthy impact on BCTQ-SSS (p=0.047) and BCTQ-total (p=0.033) scores, suggesting a potential synergistic effect on these measurements.

**Table 6:** The factors influencing BCTQ score: the results of MANOVA test ^a^ R^2^ = 0.945, ^b^ R^2 ^= 0.918, ^c^ R^2^ = 0.951 BCTQ: Boston Carpal Tunnel Syndrome Questionnaire, SSS: symptom severity scale, FSS: functional status scale, BMI: body mass index, MANOVA: multivariate analysis of variance.

Source	Dependent Variable	Type III Sum of Squares	df	Mean Square	F	p	Partial η^2^
Model	BCTQ-SSS	28383.591^a^	30	946.12	77.587	0	0.957
	BCTQ-FSS	17020.235^b^	30	567.341	51.232	0	0.936
	BCTQ-Total	89202.639^c^	30	2973.421	88.536	0	0.962
Group: dentists, controls	BCTQ-SSS	67.229	1	67.229	5.513	0.021	0.05
	BCTQ-FSS	81.744	1	81.744	7.382	0.008	0.066
	BCTQ-Total	297.236	1	297.236	8.85	0.004	0.078
Sex: women, men	BCTQ-SSS	28.351	1	28.351	2.325	0.13	0.022
	BCTQ-FSS	5.813	1	5.813	0.525	0.47	0.005
	BCTQ-Total	59.839	1	59.839	1.782	0.185	0.017
BMI (kg/m^2^): normal, overweight, obese	BCTQ-SSS	33.492	2	16.746	1.373	0.258	0.025
	BCTQ-FSS	18.348	2	9.174	0.828	0.44	0.016
	BCTQ-Total	85.007	2	42.504	1.266	0.286	0.024
Frequent object dropping: absent, present	BCTQ-SSS	1.443	1	1.443	0.118	0.732	0.001
	BCTQ-FSS	13.87	1	13.87	1.253	0.266	0.012
	BCTQ-Total	24.259	1	24.259	0.722	0.397	0.007
Total activity (h/week): ≤40hours, >40hours	BCTQ-SSS	10.308	1	10.308	0.845	0.36	0.008
	BCTQ-FSS	8.079	1	8.079	0.73	0.395	0.007
	BCTQ-Total	36.638	1	36.638	1.091	0.299	0.01
Group * Sex	BCTQ-SSS	0.159	1	0.159	0.013	0.909	0
	BCTQ-FSS	0.137	1	0.137	0.012	0.912	0
	BCTQ-Total	0.001	1	0.001	0	0.996	0
Group * BMI	BCTQ-SSS	7.633	1	7.633	0.626	0.431	0.006
	BCTQ-FSS	2.972	1	2.972	0.268	0.606	0.003
	BCTQ-Total	20.129	1	20.129	0.599	0.441	0.006
Group * Frequent object dropping	BCTQ-SSS	49.159	1	49.159	4.031	0.047	0.037
	BCTQ-FSS	30.125	1	30.125	2.72	0.102	0.025
	BCTQ-Total	156.25	1	156.25	4.652	0.033	0.042
Group * Total activity	BCTQ-SSS	0.215	1	0.215	0.018	0.895	0
	BCTQ-FSS	2.258	1	2.258	0.204	0.653	0.002
	BCTQ-Total	3.867	1	3.867	0.115	0.735	0.001
Sex * BMI	BCTQ-SSS	8.297	2	4.148	0.34	0.712	0.006
	BCTQ-FSS	21.736	2	10.868	0.981	0.378	0.018
	BCTQ-Total	56.419	2	28.21	0.84	0.435	0.016
Sex * Frequent object dropping	BCTQ-SSS	48.608	1	48.608	3.986	0.048	0.037
	BCTQ-FSS	3.699	1	3.699	0.334	0.565	0.003
	BCTQ-Total	79.126	1	79.126	2.356	0.128	0.022
Sex * Frequent object dropping	BCTQ-SSS	1.645	1	1.645	0.135	0.714	0.001
	BCTQ-FSS	0.503	1	0.503	0.045	0.832	0
	BCTQ-Total	3.968	1	3.968	0.118	0.732	0.001
BMI * Frequent object dropping	BCTQ-SSS	9.198	1	9.198	0.754	0.387	0.007
	BCTQ-FSS	2.708	1	2.708	0.245	0.622	0.002
	BCTQ-Total	1.924	1	1.924	0.057	0.811	0.001
BMI * Total activity	BCTQ-SSS	1.132	1	1.132	0.093	0.761	0.001
	BCTQ-FSS	0.385	1	0.385	0.035	0.852	0
	BCTQ-Total	0.197	1	0.197	0.006	0.939	0
Frequent object dropping * Total activity	BCTQ-SSS	2.528	1	2.528	0.207	0.65	0.002
	BCTQ-FSS	0.287	1	0.287	0.026	0.872	0
	BCTQ-Total	4.519	1	4.519	0.135	0.714	0.001
Group * Sex * BMI	BCTQ-SSS	9.021	1	9.021	0.74	0.392	0.007
	BCTQ-FSS	2.39	1	2.39	0.216	0.643	0.002
	BCTQ-Total	20.699	1	20.699	0.616	0.434	0.006
Group * Sex * Frequent object dropping	BCTQ-SSS	9.058	1	9.058	0.743	0.391	0.007
	BCTQ-FSS	0.115	1	0.115	0.01	0.919	0
	BCTQ-Total	11.213	1	11.213	0.334	0.565	0.003
Group * Sex * Total activity	BCTQ-SSS	36.86	1	36.86	3.023	0.085	0.028
	BCTQ-FSS	37.304	1	37.304	3.369	0.069	0.031
	BCTQ-Total	148.326	1	148.326	4.417	0.038	0.04
Group * BMI * Frequent object dropping	BCTQ-SSS	13.622	1	13.622	1.117	0.293	0.011
	BCTQ-FSS	20.75	1	20.75	1.874	0.174	0.018
	BCTQ-Total	67.996	1	67.996	2.025	0.158	0.019
Group * BMI * Total activity	BCTQ-SSS	3.524	1	3.524	0.289	0.592	0.003
	BCTQ-FSS	7.7	1	7.7	0.695	0.406	0.007
	BCTQ-Total	21.641	1	21.641	0.644	0.424	0.006
Group * Frequent object dropping * Total activity	BCTQ-SSS	39.203	1	39.203	3.215	0.076	0.03
	BCTQ-FSS	18.748	1	18.748	1.693	0.196	0.016
	BCTQ-Total	112.172	1	112.172	3.34	0.07	0.031
Sex * BMI * Frequent object dropping	BCTQ-SSS	0.716	1	0.716	0.059	0.809	0.001
	BCTQ-FSS	3.718	1	3.718	0.336	0.564	0.003
	BCTQ-Total	1.171	1	1.171	0.035	0.852	0
Sex * BMI * Total activity	BCTQ-SSS	3.634	1	3.634	0.298	0.586	0.003
	BCTQ-FSS	0.704	1	0.704	0.064	0.801	0.001
	BCTQ-Total	7.537	1	7.537	0.224	0.637	0.002
Sex * Frequent object dropping * Total activity	BCTQ-SSS	6.297	1	6.297	0.516	0.474	0.005
	BCTQ-FSS	9.515	1	9.515	0.859	0.356	0.008
	BCTQ-Total	31.293	1	31.293	0.932	0.337	0.009
BMI * Frequent object dropping * Total activity	BCTQ-SSS	0	0	.	.	.	0
	BCTQ-FSS	0	0	.	.	.	0
	BCTQ-Total	0	0	.	.	.	0
Group * Sex * BMI * Frequent object dropping	BCTQ-SSS	0	0	.	.	.	0
	BCTQ-FSS	0	0	.	.	.	0
	BCTQ-Total	0	0	.	.	.	0
Group* Sex * BMI * Total activity	BCTQ-SSS	2.577	1	2.577	0.211	0.647	0.002
	BCTQ-FSS	0.022	1	0.022	0.002	0.964	0
	BCTQ-Total	3.081	1	3.081	0.092	0.763	0.001
Group * Sex * Frequent object dropping * Total activity	BCTQ-SSS	0.334	1	0.334	0.027	0.869	0
	BCTQ-FSS	0.855	1	0.855	0.077	0.782	0.001
	BCTQ-Total	0.121	1	0.121	0.004	0.952	0
Group * BMI * Frequent object dropping * Total activity	BCTQ-SSS	0	0	.	.	.	0
	BCTQ-FSS	0	0	.	.	.	0
	BCTQ-Total	0	0	.	.	.	0
Sex * BMI * Frequent object dropping* Total activity	BCTQ-SSS	0	0	.	.	.	0
	BCTQ-FSS	0	0	.	.	.	0
	BCTQ-Total	0	0	.	.	.	0
Group * Sex * BMI * Frequent object dropping * Total activity	BCTQ-SSS	0	0	.	.	.	0
	BCTQ-FSS	0	0	.	.	.	0
	BCTQ-Total	0	0	.	.	.	0

## Discussion

The results of the present study suggest that in individuals belonging to a risk group with a certain occupational profession, CTS symptoms may start at an age range earlier than expected. The BCTQ scores in dentists were found to be significantly higher compared to the control group, which consists of individuals from a profession that does not carry a specific risk for CTS. Moreover, when being a dentist and experiencing frequent object dropping were considered together, it led to a significant increase in BCTQ scores. CTS is generally observed in the middle and older age groups [1,2,18], but its certain symptoms, including frequent object dropping, were also seen in dentists under the age of 40.

Pazzaglia et al. reported that the occurrence of "dropping objects" in patients with CTS appears to be an indicator of the severity of CTS [9]. In this study, our aim was to investigate whether an index for dropping objects could be used to identify individuals at risk of developing functionally mild CTS. We observed a significant correlation between the frequency of dropping objects and dentists who performed tasks involving prolonged hand usage. Around 23% of dentists reported dropping objects more than once a week. Although dentists are more prone to dropping objects due to carrying dental instruments as a routine practice, this can be considered a confounding factor. Certain professions that are more susceptible to CTS, such as helicopter pilots, may exhibit fewer instances of object dropping. This is largely due to the specific requirements and nature of their job. Therefore, the correlation between object dropping and CTS is nuanced and can vary significantly based on the profession in question. However, we still found a significant association between BCTQ scores and the frequency of dropping objects from the hand. Therefore, accidental object dropping may serve as an indicator of functionally mild CTS, even in the absence of electrophysiological findings, and should be taken into account in clinical practice.

Clinically, CTS can be diagnosed based on specific symptoms and examination findings. CTS typically manifests with symptoms such as hand paraesthesia, hypoesthesia, and in severe cases thenar atrophy and hand weakness. However, hand pain, tingling, and numbness are also commonly observed symptoms in the general population [19]. Therefore, it is important to search for the symptoms that strongly suggest CTS. These symptoms include nocturnal tingling or numbness that is relieved by hand movements, hand pain and/or tingling triggered by hand force, and sensory symptoms occurring in digits one, two, three, or half of the fourth digit, or in a combination of these digits [2]. In this study, symptomatic cases were screened in both groups using the BCTQ-SSS revealing that the number of symptomatic cases was higher among dentists, and the median BCTQ-SSS score was also higher in this group.

The neurological examination findings revealed that 18% of dentists in the study group exhibited paraesthesia in the median nerve skin innervation area. No individuals in the control group reported similar experiences. Phalen's test also yielded a higher rate of positive results in the study group compared to the controls, although it was not statistically significant. Clinical provocative tests, such as Phalen's or Tinel's test, can be helpful in confirming the diagnosis of CTS when conducted using standardized and well-defined protocols. However, it is important to note that the sensitivity of Phalen’s tests ranges from 42% to 85%, and the specificity ranges from 54% to 98% [18,19]. These findings suggest that although not definitive, certain symptoms may begin to manifest from an early age in dentists who are at risk for CTS.

While a positive history and clinical examination can strongly suggest CTS, NCSs of the bilateral median nerves are recommended for confirming the diagnosis and evaluating the severity of the disease [2]. However, it is well known that some patients with the typical symptoms of CTS can have normal NCS exams [20,21]. In this study, only the cases with high BCTQ scores were examined and one dentist showed mild electrodiagnostic evidence of CTS. While NCSs can be a valuable diagnostic tool, they may not always detect CTS accurately in its early stages or in younger patient populations. Clinicians should consider this limitation when interpreting NCS results, particularly in cases where upper limb symptoms are present but primary CTS symptoms are absent. Additional clinical evaluation and follow-up may be necessary to establish a definitive diagnosis or explore alternative causes for the observed symptoms [19].

The BCTQ is a scale developed to evaluate the severity of symptoms associated with CTS and their impact on the functional status of patients. It was specifically designed for monitoring and following up of individuals with CTS [12]. Implementing the BCTQ as a screening tool can be beneficial in identifying individuals who may be at risk for CTS and require further diagnostic evaluation. The study conducted by Sirisena et al. suggests that the BCTQ has the potential to be used as a screening tool for CTS in individuals who have not previously been diagnosed with the condition [22]. The BCTQ offers a comprehensive assessment by addressing both symptomatic and functional limitations. It allows for a detailed evaluation of symptoms and can be utilized for tracking the progression of CTS before and after treatment. In the present study, 33 out of 61 (54.1%) healthy controls who did not have professions needing excessive hand use reported symptoms or limitations in their functional status according to BCTQ. This finding suggests that the BCTQ may have relatively low specificity as a screening questionnaire for CTS. However, considering the small size of our study group, it is necessary to validate these results in much larger populations. On the other hand, this also raises the possibility that these individuals, unless they modify their hand usage habits, may be at risk of developing CTS in the later stages of their lives.

This study has certain limitations. The most significant limitation is that EMG examinations were not conducted for all participants. Although normal NCS results may be consistent with clinical findings, EMG examination is an important tool for diagnostic support and determining the severity of CTS. Additionally, it would have been beneficial to perform subgroup analyses for certain BCTQ questions that strongly suggest CTS, such as nocturnal paraesthesia. Some of the BCTQ scores in this study were derived from more non-specific questions, such as hand and wrist pain. Subgroup analyses could have helped us understand the relationship between specific symptoms and CTS more comprehensively. In this study, previously reported risk factors for CTS, such as being overweight, having a higher BMI, and being female, did not significantly impact BCTQ scores [3,4,18]. This finding may be related to the fact that most participants in our study had normal body weight and BMI. When it comes to occupational groups with a risk of developing CTS, the significance of sex may diminish.

## Conclusions

This study has provided insights into CTS and its association with occupational groups, particularly dentists. The results suggest that CTS symptoms may manifest at a younger age in individuals at higher risk due to their occupational profession. There was a clear positive correlation between CTS-related symptoms and the prolonged duration of hand usage, indicating that the repetitive movements and pressure during dental procedures can contribute to the development of CTS. The finding that accidental object dropping may serve as an indicator of functionally mild CTS is noteworthy. Although dropping objects can occur for various reasons, its frequency in dentists correlated with higher BCTQ scores, suggesting its relevance to CTS. In conclusion, this study sheds light on the early onset of CTS symptoms in high-risk occupational groups, emphasizing the need for vigilance and preventive measures. Further research and education in this area can help improve the overall well-being of dentists and other high-risk professionals, ultimately benefiting both their occupational health and patient care.

## Appendices

**Table 7:** The Turkish version of the Boston Carpal Tunnel Syndrome Questionnaire Form: Symptom Severity Scale (first), and Functional Status Scale (second). [14]

	Semptom Şiddet Skalası	1	2	3	4	5
1	Gece el veya el bileği ağrınızın derecesi nedir?	Yok	Hafif	Orta	Şiddetli	Çok şiddetli
2	Son iki hafta içinde el veya el bileği ağrısı nedeniyle bir gecede ortalama kaç defa uyandınız?	Yok	1 kez	2–3 kez	4-5 kez	5’ten fazla
3	Gündüz el veya el bileğinizde ağrınız oluyor mu?	Yok	Hafif	Orta	Şiddetli	Çok şiddetli
4	Gündüz kaç defa el veya el bileğinizde ağrınız oluyor?	Yok	1-2 kez	3-5 kez	5’ten fazla	Devamlı
5	Gündüz bir ağrı dönemi ortalama ne kadar sürüyor?	Yok	<10 dakika	10–60 dakika	>60 dakika	Devamlı
6	Elinizde hissilik (duyu kaybı) var mı?	Yok	Hafif	Orta	Ciddi	Çok ciddi
7	El veya el bileğinizde güçsüzlük var mı?	Yok	Hafif	Orta	Ciddi	Çok ciddi
8	Elinizde karıncalanma hissi oluyor mu?	Yok	Hafif	Orta	Ciddi	Çok ciddi
9	Elinizdeki his kaybı ve karıncalanma gece ne kadar şiddetli oluyor?	Yok	Hafif	Orta	Ciddi	Çok ciddi
10	Son iki hafta içinde ortalama bir gecede kaç kez elinizde his kaybı veya karıncalanma ile uyandınız?	Yok	1 kez	2–3 kez	4-5 kez	5’ten fazla
11	Anahtar veya kalem gibi küçük resimleri tutmak ve kavramakta zorluk çekiyor musunuz?	Yok	Hafif	Orta derecede	Çok	Aşırı

	Fonksiyonel Durum Skalası	Zorlanmadan	Hafif zorlanarak	Orta derecede zorlanarak	Şiddetli zorlanarak	El veya el bileği şikâyetlerim nedeniyle hiç yapamıyorum
1	Yazı yazmak	1	2	3	4	5
2	Giysilerin düğmesini iliklemek	1	2	3	4	5
3	Okurken kitabı tutmak	1	2	3	4	5
4	Telefon ahizesini tutmak	1	2	3	4	5
5	Kavanoz açmak	1	2	3	4	5
6	Alışveriş torbalarını taşımak	1	2	3	4	5
7	Günlük ev işleri	1	2	3	4	5
8	Banyo yapmak ve giyinmek	1	2	3	4	5
